# Making sense of chemical space network shows signs of criticality

**DOI:** 10.1038/s41598-023-48107-3

**Published:** 2023-12-04

**Authors:** Nicola Amoroso, Nicola Gambacorta, Fabrizio Mastrolorito, Maria Vittoria Togo, Daniela Trisciuzzi, Alfonso Monaco, Ester Pantaleo, Cosimo Damiano Altomare, Fulvio Ciriaco, Orazio Nicolotti

**Affiliations:** 1 Dipartimento di Farmacia - Scienze del Farmaco, Università degli studi di Bari Aldo Moro, via E. Orabona, 4, 70125 Bari, Italy; 2 Istituto Nazionale di Fisica Nucleare, Sezione di Bari, via E. Orabona, 4, 70125 Bari, Italy; 3 Division of Medical Genetics, Fondazione IRCCS-Casa Sollievo della Sofferenza, San Giovanni Rotondo (Foggia), Italy; 4 Dipartimento Interateneo di Fisica “M. Merlin”, Università degli studi di Bari Aldo Moro, Via Giovanni Amendola, 173, 70125 Bari, Italy; 5 Dipartimento di Chimica, Università degli studi di Bari Aldo Moro, via E. Orabona, 4, 70125 Bari, Italy

**Keywords:** Complex networks, Phase transitions and critical phenomena, Computational models, Medicinal chemistry

## Abstract

Chemical space modelling has great importance in unveiling and visualising latent information, which is critical in predictive toxicology related to drug discovery process. While the use of traditional molecular descriptors and fingerprints may suffer from the so-called curse of dimensionality, complex networks are devoid of the typical drawbacks of coordinate-based representations. Herein, we use chemical space networks (CSNs) to analyse the case of the developmental toxicity (Dev Tox), which remains a challenging endpoint for the difficulty of gathering enough reliable data despite very important for the protection of the maternal and child health. Our study proved that the Dev Tox CSN has a complex non-random organisation and can thus provide a wealth of meaningful information also for predictive purposes. At a phase transition, chemical similarities highlight well-established toxicophores, such as aryl derivatives, mostly neurotoxic hydantoins, barbiturates and amino alcohols, steroids, and volatile organic compounds ether-like chemicals, which are strongly suspected of the Dev Tox onset and can thus be employed as effective alerts for prioritising chemicals before testing.

## Introduction

The canonical representation of chemical spaces based on a coordinate system with multiple dimensions suffers from several issues. It is not invariant to the chosen representation: changing the adopted features can dramatically affect the boundaries of the chemical space and its properties. It cannot deal naturally with features that are both discrete and continuous^1,2^. In this regard, metric spaces can make things even harder while complex networks, which are intrinsically non-metric, can promptly offer a solution.

In recent years, the opportunities given by the adoption of complex networks to model the chemical spaces, the so-called chemical space networks (CSNs), have been widely investigated. Several fields have been studied, such as medicinal chemistry, physicochemical properties, and de novo drug design, just to mention a few^3–8^. An additional advantage provided by CSNs is the smart mathematical framework behind them that is the graph theory. Centrality metrics such as degree, betweenness and eigenvector centrality can suitably characterise the behaviour of the chemicals within a network, while their distribution can deepen our understanding of the network organisation and, therefore, of the resulting chemical space^9^. Topological properties allow for the characterization of a network organisation, for example the presence of hubs or communities. Features, such as scale-freeness or small-worldness, can signal the presence of patterns and dynamics within a network as extensively reported elsewhere^10,11^; by contrast, random graph models, such as the Erdos–Renyi (ER) model, can be used for benchmarking or to assess the meaningfulness of specific structures and architectures^12^.

Previous studies have investigated CSNs as threshold networks, i.e., networks whose structures depend and vary according to specific cut-off values set on the network connections. The constituent elements of these networks, usually called nodes, are chemicals while connections are pairwise molecular similarities: these studies were aimed at comparing different datasets^13–15^. This approach has shown how different similarity metrics generate different CSNs, how different choices of the similarity cut-off affect nodal properties like degree or assortativity, among the others, and the presence of molecular communities^16–18^. Defining an optimal cut-off is far from being a simple fine-tuning matter and varying the similarity threshold adopted to construct a network deeply shapes the network topology and its meaningfulness^14,19,20^. Moreover, the task is complicated by the huge heterogeneity of the chemical space. To mitigate this issue, this work will be focused on a reduced yet extremely interesting class of chemicals, consisting of small molecules experimentally labelled as toxic with respect to developmental toxicity (Dev Tox).

Herein, we investigate the possibility of choosing an optimal threshold based on statistical mechanics properties. We identify a first-order phase transition, a signal of emergent behaviours within a complex system, as a flag that an optimal cut-off has been reached. Although this perspective has been thoroughly reported in several case studies and has demonstrated its effectiveness by providing fundamental advances in our understanding of collective phenomena^21–26^, an application to the CSNs is still missing. More broadly, our goal is to provide interpretable insights on CSNs.

The data investigated deal with Dev Tox. This concerns offspring abnormal development due to the exposure to harmful agents or to hazard conditions^27^. It is a complex human health endpoint, of utmost importance especially for the care of the maternal and child health. Predicting the Dev Tox onset remains extremely challenging and far from reaching satisfactory levels of accuracy^28,29^. In the last decade, several predictive approaches, especially based on machine learning, have been proposed^30–33^.

The knowledge gap in understanding Dev Tox depends on two intimately related aspects: on one side the limited amount of Dev Tox measured data as well as their uncertainty and on the other the structural complexity of the chemicals reflecting the space heterogeneity. Hence, we propose to employ the CSN perspective to model such heterogeneity and gain fundamental insights about which toxic chemicals share common molecular patterns and, eventually, deepen our rational understanding of the latent toxicological mechanisms behind Dev Tox. In this respect, our study also falls in the broad cutting-edge domain of eXplainable Artificial Intelligence (XAI)^34–37^.

## Results

### Criticality signals optimal thresholding

To study the CSN of Dev Tox, a database of small molecules, whose toxic effects are well established, was collected. To the best of our knowledge, the data used here represent the largest publicly available base of knowledge for Dev Tox. Two main sources were taken into account for modelling Dev Tox. The CAESAR^30^ and the Procter & Gamble (P&G) datasets^38^, including 201 and 621 experimentally toxic chemicals, respectively. The Food and Drug Administration (FDA) classifies as toxic the chemicals belonging to one of the three following categories^39,40^: class C that reports chemicals tested positive for Dev Tox in animal studies; class D that reports chemicals tested positive for Dev Tox only in human studies; class X that reports chemical tested positive in both animal and human studies and/or had evidence of foetal risk based on human experience.

Preliminary examinations were carried out to select the most reliable chemicals for Dev Tox modelling and, after removing duplicates, a number N of 684 toxic chemicals were included in this study. List of all chemicals with their structures and Dev Tox annotations is available in the Table S1 of the Supporting Information. The Table S1 also includes a list of 135 non-toxic chemicals available from the previously mentioned data repository which were used for classification purposes.

**Figure 1:**
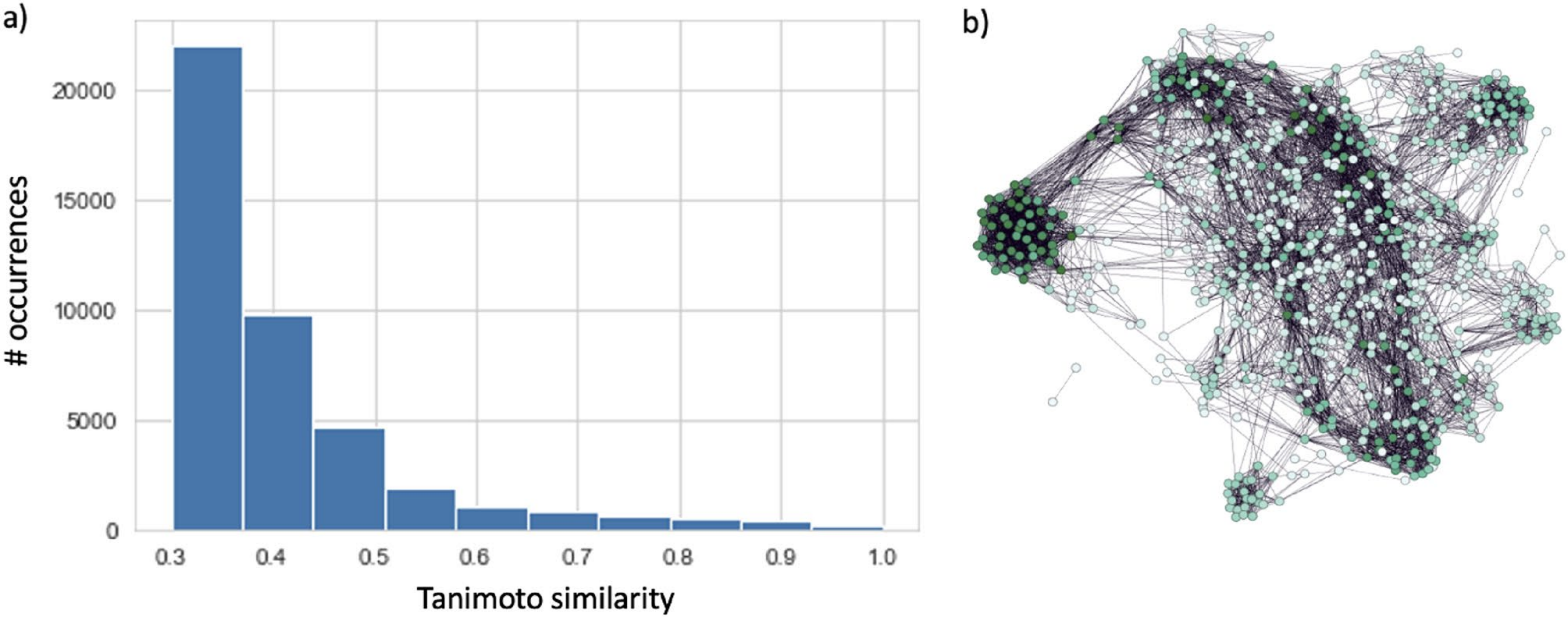
Similarity distribution of Tanimoto values (**a**). Chemical space network (**b**).

N(N-1)/2 pairwise Small Molecule Subgraph Detector (SMSD)^41^ Tanimoto^42^ similarity measures were calculated and connections between nodes with similarity greater than the cut-off value, set to 0.3, were established based on the assumption that a very low similarity does not reflect any meaningful information. Such a choice allowed a remarkable computational burden reduction. The resulting similarity distribution along with the CSN is presented in Fig. 1.

This study aims at analysing the CSN topology, thus network weights are neglected as topological features are generally weight-independent^43,44^; nevertheless, weights become crucial when considering different cut-off values and this can dramatically affect topology. Hence, by thresholding Tanimoto similarities at different cut-off values, we investigated the variations occurring within the CSN in terms of three main centrality metrics: degree (d), betweenness (b) and eigenvector centrality (e). Moreover, a paired analysis on an ensemble of ER graphs comparable with the CSN was carried out.

For each threshold value, the number of CSN edges $E$ was computed with the maximum possible value being:

$${E}_{max}=N\left(N-1\right)/2$$

with N, the number of chemicals, being the CSN order.

**Figure 2:**
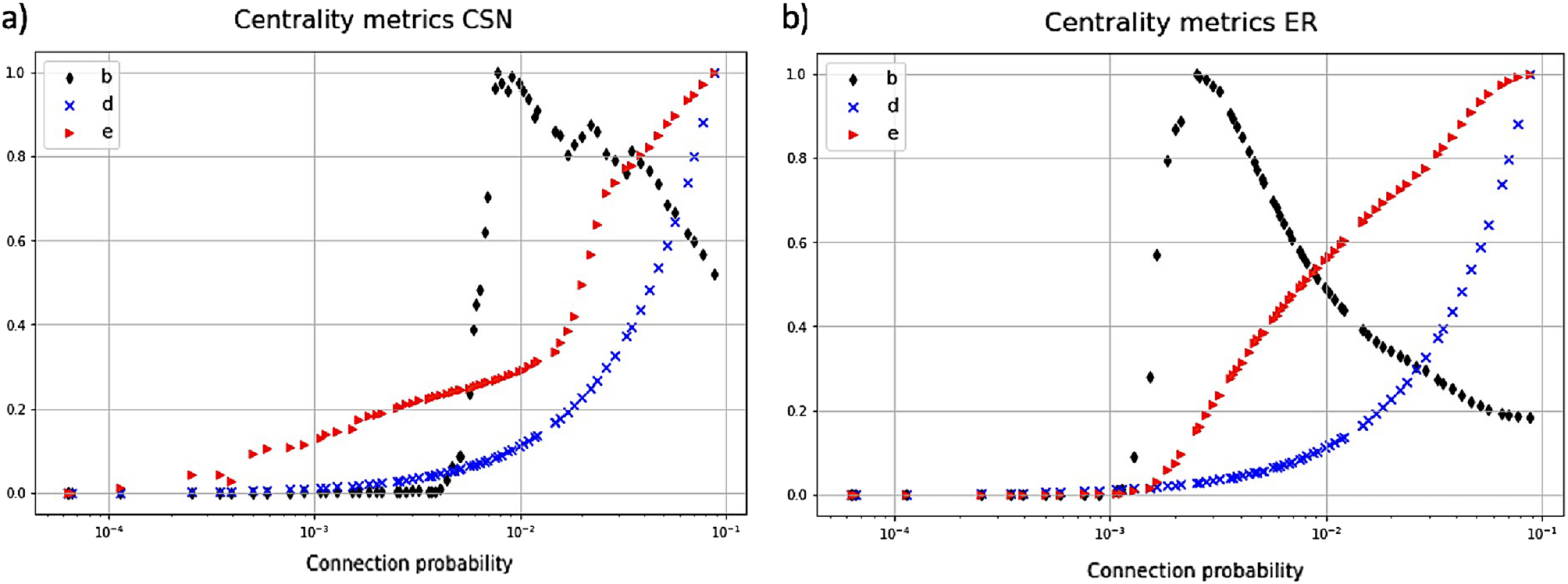
Centrality metrics as a function of the Tanimoto similarity for both (**a**) CSN and (**b**) ER networks. Betweenness is represented in black diamonds, degree with blue crosses and eigenvector centrality with red triangles. Centrality measures are scaled in the [0,1] interval for ease of comparison.

Thus, the connection probability

$$p=E/{E}_{max}$$

was calculated and an ER model $G\left(N,p\right)$ was simulated (for statistical robustness, 20 different simulations were performed). The results are shown in Fig. 2.

The number of CSN connections occurring in the model ranges from 0 (when the Tanimoto threshold is 1) to 41,807 (when the Tanimoto threshold is 0.3) while the resulting connection probability *p* ranges approximately within $0\div {10}^{-1}$ which includes the ER critical probability, which is ${p}_{crit} \sim 1/N={10}^{-3}$. As expected, centrality metrics intrinsically related to edge counting, such as degree and eigenvector centrality, showed monotonically decreasing trends. This can easily be explained as a direct consequence of the edge removal. Apart from statistical fluctuations, the degree is equal for both models. In fact, by construction, the ER model is simulated with the connection probability retrieved by the CSN. Eigenvector centrality has higher values in the ER ensemble, while for higher probability values the two trends are substantially similar. An analogous consideration holds for eigenvector centrality and betweenness (see Fig. S1 of the Supporting Information for a detailed comparison). For both CSN and ER models, betweenness increases with connection probability until a maximum is reached, then betweenness abruptly decreases. Interestingly, while this phase transition occurs as expected with $p \sim {p}_{crit}$ in the ER model, for the CSN model this phase transition occurs at a slightly higher value ${p}_{crit}^{CSN} {\sim 5\cdot 10}^{-3}$ which corresponds to a Tanimoto similarity of ~ 0.7. Overall, these results highlight the presence of an optimal cut-off signalled by the behaviour of betweenness and a problematic similarity between the constructed CSN and a random graph.

### The CSN is not random

The CSN being a random graph would be not only far from intuition, as chemicals showing similar behaviours should be close in the chemical space, but it would also pose fundamental issues as a random network by definition does not include meaningful structures.

**Figure 3:**
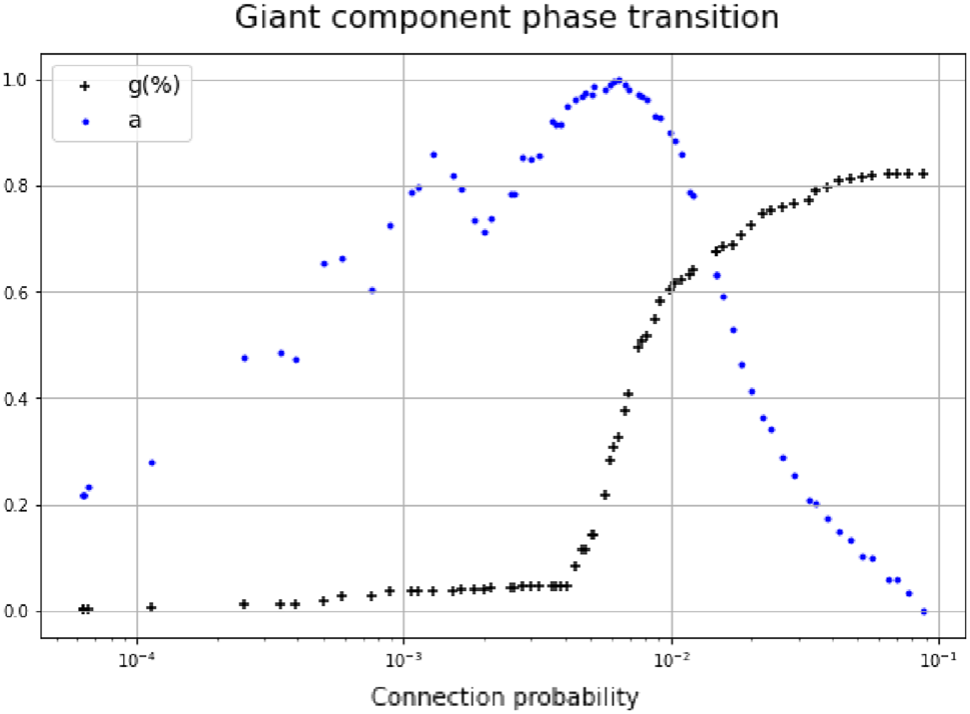
Giant component phase transition. The percentage of nodes within the giant component (black crosses) and the normalised assortativity (blue points) are shown as functions of connection probability.

It can be easily shown that the phase transition signalled by the peak in betweenness corresponds to the first-order phase-transition of the giant component in a random graph, although it does not occur at $p 1/N$, see Fig. 3. The fraction of nodes within the giant component becomes non-null at the same critical probability ${p}_{crit}$ at which betweenness abruptly increases (as previously shown in Fig. 2). However, the studied CSN cannot be a random graph and the definitive proof is provided by assortativity. In fact, Fig. 3 shows that assortativity increases with the connection probability until it reaches a maximum at ${p}_{crit}$.

This behaviour is a consequence of the transitivity of Tanimoto similarity. Low probabilities correspond to high similarity; therefore, if two chemicals are connected to a third one then they will probably be connected. This is a typical assortative behaviour, and it is not consistent with a random graph whose assortativity should be close to zero. In fact, the CSN assortativity dramatically drops, when more and more edges are added, tending to the behaviour of a random graph. Thus, despite thresholding the CSN at criticality yielded the emergence of a giant component, the meaningfulness of CSN inner structures, based on molecular similarity, is preserved.

### The Dev Tox archetypes

**Figure 4:**
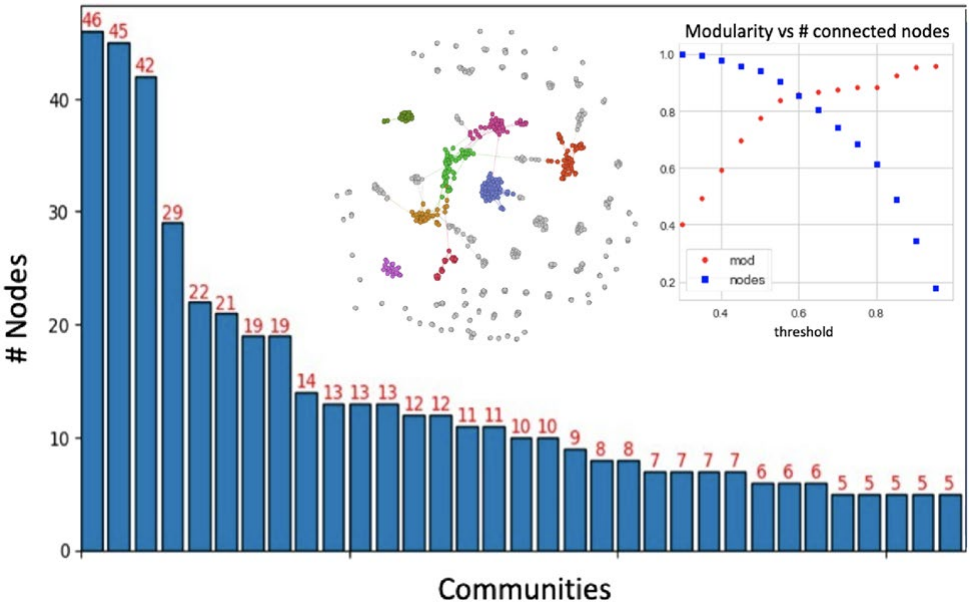
CSN at criticality: the largest 8 communities are outlined with different colours. The network nodes at criticality are basically scattered among several communities, the first 30 communities are shown here. The panel confirms that at criticality the fraction of isolated nodes is reasonable while the partition quality, in terms of modularity, reaches more than satisfactory levels.

At criticality, the CSN experiences the giant component formation while satellite groups of few chemicals are also present. A community detection analysis was performed along with a modularity analysis to highlight the inner CSN organisation; moreover, the community cardinality was examined, see Fig. 4.

At criticality, almost 25% of network nodes are grouped within the top three populated communities, consisting of 46, 45 and 42 elements. The overall modularity and the percentage of connected nodes exceeded 80%. These findings suggest the existence of specific communities, based on molecular patterns, that we will call Dev Tox “archetypes”. It should be noted that these archetypes should not be strictly intended as toxicological classes; in fact, by construction, they are based only on structural similarities of toxicophores evaluated by means of the Tanimoto metric. To gain further insights into the chemical meaning of these communities, we computed hundreds of molecular descriptors (from physicochemical to auto-correlation properties) for each toxicophore and investigated their statistical association within the archetypes. After Bonferroni correction, we found 145 descriptors whose distributions can be significantly distinguished, at 1% significance, within the three communities.

This analysis highlighted the presence of descriptors capturing basic and easy-to-interpret features such as molecular weight, number of valence electrons and molecular refractivity. Interestingly, such descriptors are of particular interest when evaluating properties of fundamental importance such as drug-likeness^45,46^. Other descriptors significantly related to communities were well-known topochemical indices such as BCUT descriptors, BertzCT and molecular connectivity chi indexes^47^. Finally, an extremely relevant role was played by Moreau-Broto autocorrelation descriptors^48^. Topological autocorrelation is frequently used in Quantitative Structure–Activity Relationship (QSAR) models^49^ to assess how specific physicochemical properties are spatially distributed along molecules. Here, almost 50% of significant descriptors consisted of autocorrelation patterns, specifically involved with (i) atomic properties (number of valence or sigma electrons), mass, atomic numbers; (ii) electronegativity (Sanderson, Pauling and Allred-Rochow)^50^.

**Figure 5:**
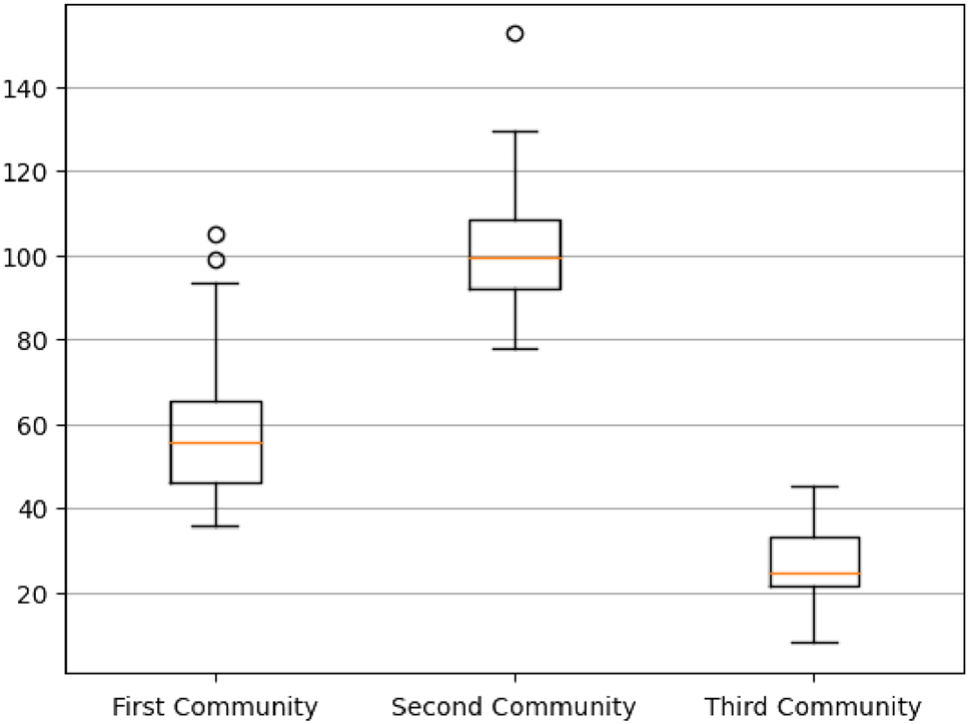
The boxplot shows the variability range for the molecular refractivity within the top three populated communities.

Besides statistical significance, the median value for each descriptor and its interquartile range were evaluated to highlight the different behaviour within each community along with its variability. For example, the molecular refractivity is shown in Fig. 5.

A comprehensive list of significant descriptors and their characteristic ranges for Dev Tox is provided in the Table S2 of the Supporting Information. These results confirm that the communities reflect structural similarities and therefore they include chemicals with different properties.

**Figure 6:**
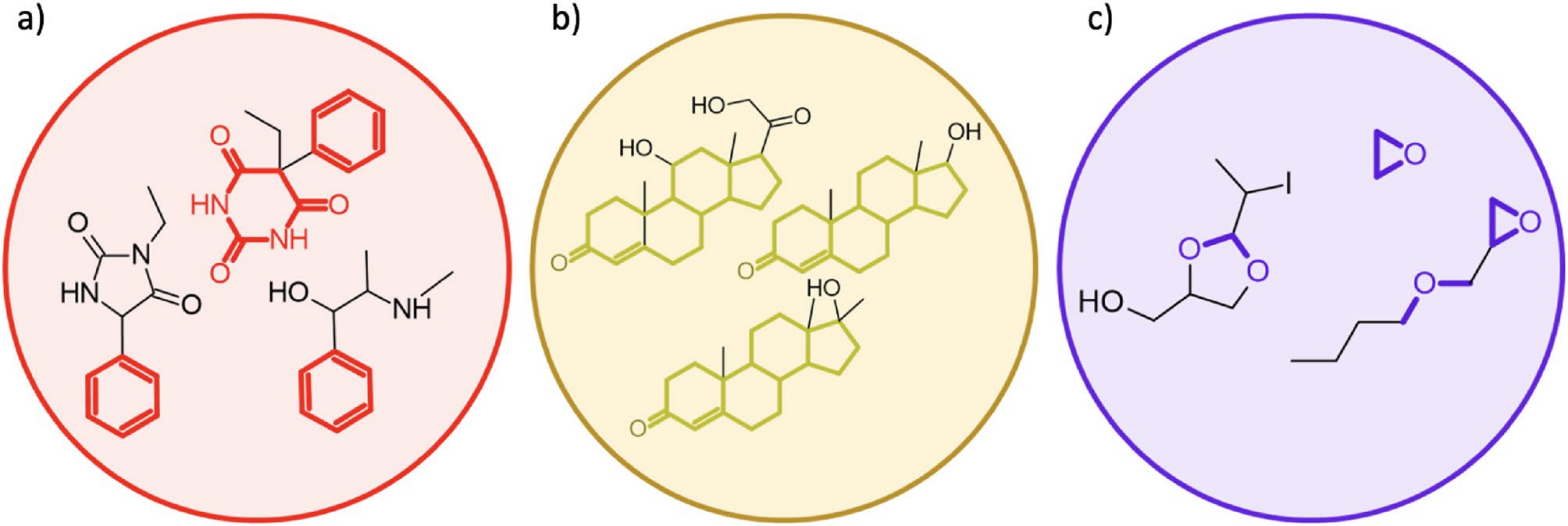
The most representative chemical structures of the three top communities: (**a**) aryl derivatives mainly comprising barbiturates and amino alcohols; (**b**) cyclopentanoperhydrophenanthrene cores typical of the steroid lipid family; (**c**) small Volatile Organic Compounds (VOCs) ether-like chemicals.

In particular, the first community includes heterogeneous toxicophores in terms of structural moieties, being this probably due to its large size. In general, it includes aryl derivatives mainly comprising barbiturates, hydantoins and amino alcohols, commonly used as anticonvulsant drugs, GABA modulators, excitatory amino acid antagonists, hypnotic and sedative drugs, see Fig. 6a. The second community cover toxicophores with well-known cyclopentanoperhydrophenanthrene cores typical of steroids, responsible of essential biological functions such as fluidity and permeability regulation also known for fertility impairment, see Fig. 6b. The third community is made by small Volatile Organic Compounds (VOCs) ether-like chemicals, which act as pollutants and food toxins, see Fig. 6c.

Finally, we explored whether the toxicophores within a community shared specific drug targets generally consisting of receptor and enzyme proteins. To this aim, we used the PLATO platform^51^, which is specifically aimed at pairing biological targets to small molecules on the basis of the calculated similarity with respect to known ligands annotated with experimental biological measures retrieved from the CHEMBL database. Based on highly occurring targets, we found that the first community engage mostly targets relevant for the central nervous system, normally engaged by hypnotic, sedative and anticonvulsant drugs**.** While the community of steroids mainly interplayed with the hormonal system including mineralocorticoid and glucocorticoid receptors, progesterone receptors and androgen-binding protein receptors. The third community, mostly composed of VOCs ethers-like chemicals, did not pair with any verified target and this could be due to their low similarity to known drugs.

### The CSN predictive power

**Figure 7:**
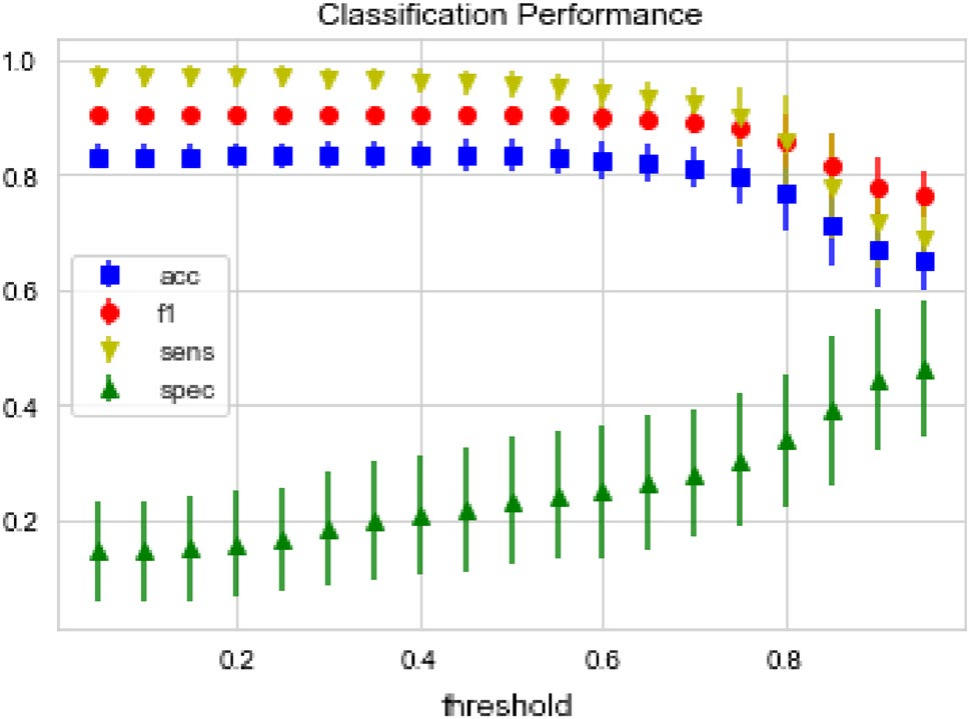
Classification metrics for CSN predictive power as a function of the Tanimoto similarity. At the preferred Tanimoto similarity threshold of ~ 0.7, the model achieves a good overall accuracy (~ 80%) with great sensitivity (> 90%) but poor specificity (~ 25%).

To evaluate the potential of CSN as a support for predictive investigations, a further analysis was carried out. We included in the CSN the nodes representing the non-toxic chemicals listed in Table S1 and designed a simple classification framework to assess to what extent the CSN is able to distinguish toxic from non-toxic chemicals. For each node/chemical to be classified, the connected nodes/chemicals were inspected along with their pairwise Tanimoto similarities; then, the classification score was computed with a weighted average. The adopted weights were the computed similarities so that the most similar chemicals were the most influential in classification. Classification results in terms of accuracy, sensitivity, specificity and f1 metrics are presented in Fig. 7.

For the sake of completeness, the classification results explored the whole range of possible Tanimoto similarity thresholds. At the critical threshold (~ 0.7), the model was able to achieve a good overall accuracy (~ 80%) and f1 (~ 90%) an extremely high sensitivity (> 90%) while specificity was significantly lower (~ 25%). Performance uncertainties were obtained by means of an 80% hold-out cross-validation, iterated 100 times.

## Discussion

The study of the chemical space is fundamentally based on one basic assumption: the structure of chemicals yields information about its physicochemical and biological properties, including the pharmacological or toxicological behaviour^52–56^. Although this paradigm is generally accepted and it has been verified for several applications, some important issues still remain not completely solved^56–61^. A fundamental limitation to this perspective concerns the extreme variability of physicochemical properties even when few or just one atomic variation occurs within a molecular structure. Hence, a potentially disrupting consideration arises: if even a subtle molecular change can turn a toxic chemical into a non-toxic chemical, then any conclusion drawn from molecular similarity is uninformative, the chemical space network and its inner communities are meaningless. A corollary question, which would prevent any possible further consideration, concerns the possibility of uniquely defining a scale at which to investigate the chemical space, i.e., to identify a suitable threshold for molecular similarity measurements.

Here, we provide an answer to both questions and demonstrate how they are indissolubly related. In fact, our findings showed an outstanding overlap between the Dev Tox CSN and an ER model; this was observed at all the threshold values, thus dangerously suggesting the possibility that the CSN was actually random. Also, the Dev Tox CSN showed a giant component phase transition as expected from an ER model. However, by definition, a random network should not show any kind of assortative behaviour: the assortativity of the Dev Tox CSN incontrovertibly demonstrates that it is not a random network, at least in the connection probability range explored. Moreover, its behaviour is far from that of a random network as the cut-off values approaches criticality. Thus, thresholding becomes intrinsically related to meaningfulness.

Once established the meaningfulness of the Dev Tox CSN, we characterised its inner communities using both molecular descriptors, as similar chemicals in a physical, biological or toxicological sense tend to exhibit similar properties^62–65^, and biological functions. The proposed CSN is thus easily interpretable by domain experts^34,66^ and could be profitably employed for drug repurposing and rational de novo design^67^, strategic assets to mitigate the well-known issues of drug discovery, such as huge costs and extremely time-consuming procedures^68–70^. In fact, thanks to PLATO target profiling, the Dev Tox archetypes outlined within the CSN could be related (with different reliability) to multiple biological activities.

This work also investigates the predictive power of the proposed CSN. The model was able to reach a reliable accuracy in Dev Tox prediction, with extremely high sensitivity. On the contrary, specificity remained substantially low. Two aspects deserve to be considered: (i) the informative content provided by structural similarity cannot reasonably provide a comprehensive description of toxicological patterns. It is easy to find examples of chemicals characterised by high structural similarity which show opposite toxicological behaviours, e.g., Dydrogesterone and Progesterone, non-toxic and toxic, respectively, differ by only a double bond^56^. (ii) While toxicants are expected to have common characteristics, the wide spectrum of chemicals which are non-toxic with respect to this specific endpoint include very heterogeneous chemotypes, which in principle can share few or even no structural similarities. Thus, for a classification model, the correct detection of non-toxic chemicals is extremely challenging, not to mention the fact that this class is poorly represented in the available databases. It is worth mentioning that this is not an unexpected behaviour, it has been already observed in literature, although by studies based on different descriptions^31–33,71–73^.

As a final remark, it is worth noting how complex network software suites and methodologies can manage systems with millions of nodes and therefore, thanks to its generality, the proposed approach can be straightforwardly adopted for broader chemical spaces, not necessarily limited to a single endpoint, and devoted to several applications. Our findings suggest that the characterization of the CSN could support in silico assessment of chemicals, specifically the so-called New Approach Methodologies (NAMs). The combined use of features derived from the CSN along with physicochemical descriptors and fingerprints could in principle enhance existing models.

## Methods

### Data curation

The chemicals were downloaded in SMILES format, with the associated binary Dev Tox label, from the freely accessible CAESAR and P&G databases. All SMILES data were cleaned of stereoisomeric assignments, were canonised and then the two databases were cross merged. 8 matches with opposite labels, derived mainly from P&G dataset, were discarded from the analysis; finally, 684 toxic molecules were collected. The selected chemicals were described by 2D molecular descriptors obtained from RDKit, and autocorrelators obtained from Mordred. Descriptors with a variability lower than 10% were removed from the analysis, thus resulting in a total of 774 descriptors, see the Table S3 of the Supporting Information for a comprehensive list.

### Network analyses

The SMSD computes the largest common subgraph between two chemicals, where the molecular graph is a natural representation of a molecule based on its bonds and its atoms, except for hydrogen atoms, which are treated as implicit. The SMSD Tanimoto measure is therefore the ratio of the size of the common subgraph between two molecule and the size of the union of in common and not in common subgraphs. We used the implementation based on current CDK available at https://mvnrepository.com/artifact/gov.nih.ncats/smsd-core.

The CSN was compared with a uniform ER model, specifically an ER model G(N,E) whose E edges are uniformly sampled among the N(N-1)/2 possible connections. The advantage of such model is to provide a graph with the same number of connections as the thresholded CSN.

Comparisons were carried out by considering three centrality metrics, accounting for three different perspectives: a local, a global and a dynamic one.

- Degree ${d}_{i}$ of node i (local metric). ${d}_{i}={\sum }_{j=1}^{N}{a}_{ij}$ with ${a}_{ij}$ representing the elements of the adjacency matrix of the considered network having N nodes. The degree takes into account only the connections of a node, in this sense it is a local centrality metric.
- Eigenvector centrality ${e}_{i}$ of node i (global metric). ${e}_{i}=\left(\frac{1}{\lambda }\right){\sum }_{j=1}^{N}{a}_{ij}{e}_{j}$ so that ${e}_{i}$ satisfies an eigenvalue equation.
- Betweenness ${b}_{i}$ of node i (dynamic metric). ${b}_{i}={\sum }_{s\ne i\ne t}^{N}\frac{{p\left(i\right)}_{st}}{{p}_{st}}$, which measures the ratio between the number of paths p connecting a generic pair of nodes (s,t) passing through the node i and all the paths connecting them. Thus, this centrality metric evaluates the dynamical information flow within the network.

Moreover, to emphasise the difference between the defined CSN and a random network, assortativity was used.

- Assortativity measures the preference of nodes to be connected to other nodes according to a similarity criterion, e.g., degree. For example, in assortative networks, high degree nodes tend to connect to nodes with high degree. On the other hand, if they prefer low-degree nodes, the network is anti-assortative. Random networks tend to have null assortativity as a consequence of connections’ randomness.

Studying these metrics allowed us to reveal the giant component phase transition. At criticality, community detection was performed by means of the Louvain algorithm^74^. All network analyses were carried out with a Python 3.8.13 distribution and the NetworkX 3.1 package.

### Statistical significance

We evaluated the differences between the distributions of several molecular descriptors within the communities that arose at criticality. To this aim, to discard any a priori hypothesis about the descriptor distributions, the non-parametric Mood’s test for medians was performed. Three tests were carried out for each descriptor to ensure that a significant difference had been found among all the three communities. The chosen significance level was 0.01 but a Bonferroni correction was adopted to avoid the multiple comparison bias.

### The PLATO platform for target profiling

The predictive web platform PLATO was used to predict relevant therapeutic drug targets of small molecules. PLATO matches query molecules with the most similar molecules in its database of experimental activity values based on Tanimoto similarity calculated on 13 different fingerprints. The predicted targets with their referenced organism are experimentally linked to the similar molecules identified by the algorithm. For each prediction, a score is calculated by summing the Tanimoto coefficients of each fingerprint. This implies that an exact match corresponds to a score equal to 13. Query reports can be easily provided in *json* format upon programmatic POST requests. PLATO is freely available at https://prometheus.farmacia.uniba.it/plato/.

### Supplementary Information

Supplementary Figure S1.

Supplementary Table S1.

Supplementary Table S2.

Supplementary Table S3.

Supplementary Legends.

## Data Availability

Data used in this work are publicly available. The entire list of Dev Tox chemicals (in SMILES format) herein analysed is provided in the Table S1 of the Supporting Information.
