# Supplementary material for: Making sense of chemical space network shows signs of criticality
Source: Sci Rep. 2023 Dec 4;13:21335. doi: 10.1038/s41598-023-48107-3 (PMC10696027; doi:10.1038/s41598-023-48107-3)
Supplement: Supplementary file 1 — Supplementary Figure S1. [file 41598_2023_48107_MOESM1_ESM.pdf]

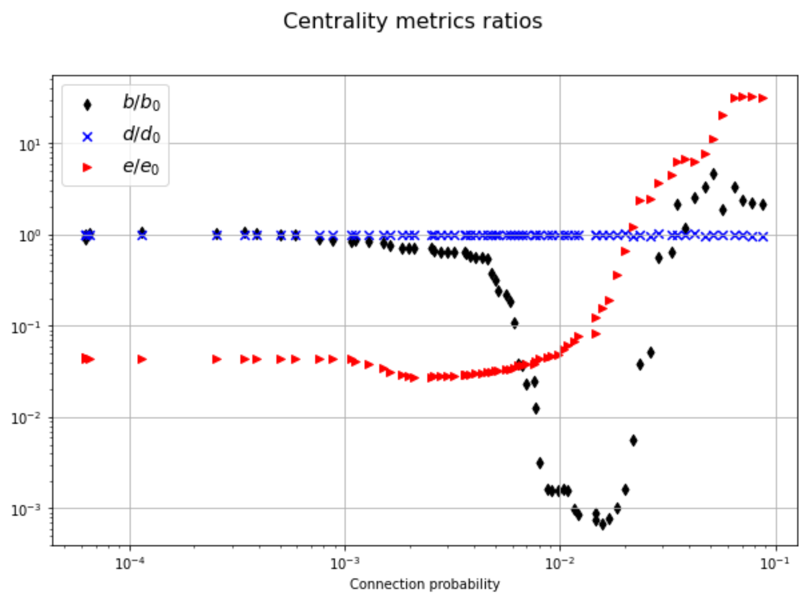

Supplementary Figure 1 | Betweenness (b), degree (d) and eigenvector centrality (e) ratios varying with the Tanimoto similarity thresholds. Ratios are computed against the ensemble averages of ER random graphs.
