# Supplementary material for: Making sense of chemical space network shows signs of criticality
Source: Sci Rep. 2023 Dec 4;13:21335. doi: 10.1038/s41598-023-48107-3 (PMC10696027; doi:10.1038/s41598-023-48107-3)
Supplement: Supplementary file 5 — Supplementary Legends. [file 41598_2023_48107_MOESM5_ESM.docx]

Supporting Information

Making sense of chemical space network shows signs of criticality

Nicola Amoroso, Nicola Gambacorta, Fabrizio Mastrolorito, Maria Vittoria Togo, Daniela Trisciuzzi, Alfonso Monaco, Ester Pantaleo, Cosimo Damiano Altomare, Fulvio Ciriaco*, Orazio Nicolotti

# Supplementary Tables

Supplementary Table 1 | List of chemicals (in SMILES format) paired with their binary Dev Tox label and modularity class. The top 3 populated communities of toxic compounds correspond to modularity class 20, 7 and 51, respectively. Non-toxic compounds and isolated nodes were not assigned to modularity class.

TableS1.csv

Supplementary Table 2 | List of 145 significant descriptors and their description. For each descriptor the median value and the interquartile range (in parenthesis) are shown the top 3 populated communities.

TableS2.csv

Supplementary Table 3 | List of 774 descriptors, paired with their source chemoinformatic library, obtained after remotion of columns with a variability lower than 10%.

TableS3.csv

# Supplementary Figures

Supplementary Figure 1 | Betweenness (b), degree (d) and eigenvector centrality (e) ratios varying with the Tanimoto similarity thresholds. Ratios are computed against the ensemble averages of ER random graphs.

FigureS1.pdf
